# A Genotype-Phenotype Analysis of Usher Syndrome in Puerto Rico: A Case Series

**DOI:** 10.7759/cureus.28213

**Published:** 2022-08-20

**Authors:** David F Santos, Leonardo J Molina Thurin, José Gustavo Vargas, Natalio J Izquierdo, Armando Oliver

**Affiliations:** 1 Department of Ophthalmology, School of Medicine, Medical Sciences Campus, University of Puerto Rico, San Juan, PRI; 2 Department of Medical Education, San Juan Bautista School of Medicine, Caguas, PRI; 3 Department of Ophthalmology, Johns Hopkins University School of Medicine, Baltimore, USA; 4 Department of Surgery, School of Medicine, Medical Sciences Campus, University of Puerto Rico, San Juan, PRI

**Keywords:** compound heterozygotes, genotype-phenotype correlation, usher syndrome, inherited retinal eye diseases, retinitis pigmentosa

## Abstract

Introduction

Patients with Usher syndrome (USH) have retinitis pigmentosa (RP) and hearing loss inherited as an autosomal recessive (ar) trait. Mutations in the *USH2A* gene are the most common cause of Usher syndrome. We report the genotype-phenotype correlation in 10 patients with Usher syndrome from Puerto Rico (PR). This is the first genotype-phenotype analysis of patients with the syndrome in PR.

Methods

We conducted a chart review of patients who carried an Usher syndrome diagnosis. They underwent a comprehensive ophthalmic evaluation by at least one of the authors. This included best corrected visual acuity (BCVA), visual field mean deviation (VF MD), pattern standard deviation (PSD), and macular optical coherence tomography (mOCT) average volume and thickness. Genotyping was done using the Invitae Inherited Retinal Disease (IRD) Panel.

Results

Three patients had a logMAR BCVA of 1.0 or worse. The median VF MD was -29.7 dB and -29.2 dB in the OD and OS, respectively. The median PSD was 5.5 dB and 5.7 dB in the OD and OS, respectively. Upon macular OCT, patients had a median volume of 8.4 μm^3^ and 8 μm^3^ in the OD and OS, respectively. The median thickness was 235 μm and 223 μm in the OD and OS, respectively. All patients had pathogenic *USH2A* variants, and eight of these were compound heterozygotes. The most common variants were p.Cys575Tyr and p.Glu767Serfs*21, each present in four patients. Patients with the p.Cys759Phe variant had the worst phenotype with the worst BCVA, largest VF MD, and slimmer macular thickness.

Conclusion

Our findings are compatible with previously reported pathogenic mutations in the *USH2A* gene. However, the p.Cys759Phe variant has previously been correlated with a mild phenotype. In our study, the p.Cys759Phe variant correlated with the most severe phenotype. This variant has a high prevalence in the Spanish population, and PR was a Spanish colony for 400 years. The presence of this variant could be traced back to Spain. Genotyping patients with Usher syndrome is of utmost importance. Further studies to evaluate the common founder effect of patients with the syndrome in PR are warranted.

## Introduction

Retinitis pigmentosa (RP) is a heterogeneous group of inherited disorders caused by the loss of photoreceptors and characterized by bony spicules appearing at the retinal mid-periphery [1,2]. RP has a classic triad of bone spicule pigmentation, attenuation of retinal vessels, and waxy pallor of the optic nerve [3]. Patients usually have symptoms of nyctalopia, followed by loss of peripheral vision [4]. Most cases progress to complete blindness [2]. RP is usually isolated but may also be associated with other systemic manifestations, the so-called syndromic retinitis pigmentosa, such as Usher syndrome (USH) [1,2].

USH is the leading cause of combined hearing and vision loss with an estimated prevalence of 1-4 per 25,000 [5]. It is inherited as an autosomal recessive (ar) trait [5]. Patients have symptoms such as vision loss, hearing loss, and occasionally vestibular dysfunction [5,6]. USH can be classified into three main types based on the degree of hearing impairment, age of RP diagnosis, and the presence of vestibular dysfunction [6]. These are Usher syndrome type I (USH1), type II (USH2), and type III (USH3) [6]. USH2 is the most common type and is estimated to affect one in 17,000 [7]. USH2 is characterized by congenital moderate to severe nonprogressive hearing loss, normal vestibular function, and the onset of RP within the second decade of life [5,6]. USH2 is usually due to mutations in the *USH2A* gene, which are also a frequent cause of nonsyndromic RP [6]. *USH2A* gene mutations account for about 80% of USH2 cases [7,8].

Previous studies have analyzed the genotype-phenotype correlation of mutations that cause Usher syndrome in other countries such as Spain, China, Canada, and the United Kingdom [5-8]. However, no studies have examined this relationship in Puerto Rico (PR). This analysis could yield information on less frequently reported mutations or reaffirm the presence of previously reported mutations. Puerto Rico also has a strong influence of Spanish and African ancestry and a history of consanguinity. Therefore, this analysis could identify common founder mutations that can be traced to other populations. Finally, a phenotype analysis can help elucidate what symptoms patients on the Island and elsewhere may experience.

We aim to identify genetic variants and evaluate how RP presents in Puerto Rican patients with Usher syndrome. We hope to establish a genotype-phenotype correlation. Establishing this correlation can lead to a more accurate understanding of the clinical course of patients with Usher syndrome. We hope that it will also improve the knowledge and awareness of Usher syndrome in PR.

## Materials and methods

We conducted a nonconcurrent prospective study on the genotype of 241 patients with a clinical diagnosis of RP in Puerto Rico. This same cohort and its data have been used in a previous study by our research team [9]. The study was approved by the Institutional Review Board of the Medical Sciences Campus of the University of Puerto Rico (B1960120).

Patients were recruited at the University of Puerto Rico during an RP symposium that took place on November 8, 2019. Saliva samples were obtained from patients who agreed to participate. All patients were Puerto Rican and consented to participate by signing a written form. Any participant younger than 18 was required to provide consent through a parent or legal guardian.

Saliva samples were screened for genetic mutations using a genotyping microarray from the Invitae Corporation. The Invitae Corporation is a laboratory that does full gene sequencing and employs next-generation sequencing (NGS) technology to identify mutations. Samples were analyzed using an Inherited Retinal Disease (IRD) Panel, which analyzes a total of 330 genes.

Patient data and panel results were collected and included in an Microsoft Excel table (Microsoft Corp., Redmond, WA, USA). This included demographic data, including township, age, and gender, as well as mutations identified. This data was evaluated to determine which patients had mutations identified as pathogenic for Usher syndrome.

Inclusion criteria included carrying a clinical diagnosis of RP, nyctalopia, decreased visual acuity (VA), and retinal findings; a visual field test and electroretinography results compatible with RP; patients with homozygous pathogenic variants in autosomal recessive (ar) genes or compound heterozygous pathogenic variants in ar genes identified as pathogenic for Usher syndrome; patients at least 18 years of age; and patients who had been evaluated by at least one of the authors.

Exclusion criteria included not being able to provide a saliva sample for genetic testing, patients with congenital nystagmus, and patients with mutations that were not associated with Usher syndrome.

The genotype of patients who met the inclusion criteria was defined. These patients underwent a comprehensive ophthalmic evaluation by at least one of the authors. This included visual acuity (VA), visual field mean deviation (VF MD) and pattern standard deviation (PSD), and macular optical coherence tomography (mOCT) average volume and thickness. Genotypic data and comprehensive evaluation results were included in a Microsoft Excel table.

## Results

Patients

A total of 13 patients met the inclusion criteria. Of these, there were seven (53.8%) female patients and six (46.2%) male patients. Their ages ranged from 33 to 77 years (average age: 52 years).

Ten (76.9%) patients had mutations in the *USH2A* gene. Of the remaining three patients, one (7.7%) had a mutation in the *USH1C* gene, one (7.7%) in the *ADGRV1* gene, and one (7.7%) in the *CEP250* gene. We chose to focus on patients with a *USH2A* gene mutation due to the low sample size of other genes.

Clinical manifestations

A total of 10 patients with *USH2A* gene mutations were included. Demographic data and clinical results for all patients included are reported in Table 1. The median values are reported in Table 2.

**Table 1:** Patient demographics and clinical features *Values are not available for all parameters.

ID	Age	Sex	Gene	VA		VF MD		VF PSD		mOCT volume		mOCT average thickness	
				OD	OS	OD	OS	OD	OS	OD	OS	OD	OS
1	22	Male	USH2A	0.2	0.2	-26.06	-27.24	7.55	7.63	8.4	8.1	234	225
2	22	Male	USH2A	0.1	0.1	-26.11	-26.83	6.01	6.03	8.5	7.9	236	221
3	61	Male	USH2A	0.4	1.3					9.2	9.3	256	259
4	51	Female	USH2A	0.3	0.3	-29.89	-30.08	5.49	5.34				
5	38	Female	USH2A	0.2	0.2	-31	-30.77	5.27	5.74	5.637	5.735	206	201
6	65	Male	USH2A			-19.85	-19.04	12.77	12.68	8.313	8.088	435	388
7	73	Female	USH2A	0.2	0.2	-29.74	-29.2	4.19	4.24	7.3	7.9	204	221
8	63	Female	USH2A	2.9	2.7								
9	33	Male	USH2A	0.1	0.1					9.3	9.1	258	253
10	64	Female	USH2A	2.8	0.4	-31.6	-30.6	2.26	3.75	4.93	5.3	170	126

**Table 2:** Median values from ophthalmic evaluation VA, visual acuity; VF MD, visual field mean deviation; VF PSD, visual field pattern standard deviation; mOCT, macular optical coherence tomography

Parameters (number, %)	OD median (IQR)	OS median (IQR)
VA (9, 90%)	0.2 (1.5)	0.2 (0.7)
VF MD (7, 70%)	-29.7 (4.9)	-29.2 (3.8)
VF PSD (7, 70%)	5.5 (3.4)	5.7 (3.4)
mOCT volume (8, 80%)	8.4 (2.4)	8 (1.8)
mOCT thickness (8, 80%)	235 (52)	223 (45)

Best corrected visual acuity (BCVA) ranged from 0.1 to 2.9. Three patients had a logMAR BCVA of 1.0 or worse. Upon Humphrey visual field testing, the mean deviation ranged from -19.85 dB to -31.6 dB. Pattern standard deviation ranged from 2.26 dB to 12.77 dB. Upon Zeiss optical coherence tomography (OCT), macular volume ranged from 4.93 μm^3^ to 9.3 μm^3^. The average macular OCT thickness ranged from 126 μm to 435 μm.

Genotype

As depicted in Table 3, all patients had pathogenic *USH2A* gene variants. Two pathogenic variants were most frequent (four patients each): p.Cys575Tyr and p.Glu767Serfs*21. All patients with the p.Cys575Tyr and p.Glu767Serfs*21 mutation were heterozygous. The next most common variants were p.Asn405Ilefs*3 and p.Cys759Phe, each found in three patients. With regard to the abovementioned mutations, two patients were heterozygous, and one was homozygous.

**Table 3:** USH2A pathogenic variants identified

Patient ID	Gene	Zygosity	Variant
1	*USH2A*	Heterozygous	c.2299del (p.Glu767Serfs*21)
1	*USH2A*	Heterozygous	c.1724G>A (p.Cys575Tyr)
2	*USH2A*	Heterozygous	c.2299del (p.Glu767Serfs*21)
2	*USH2A*	Heterozygous	c.1724G>A (p.Cys575Tyr)
3	*USH2A*	Heterozygous	c.1214del (p.Asn405Ilefs*3)
3	*USH2A*	Heterozygous	c.1724G>A (p.Cys575Tyr)
4	*USH2A*	Homozygous	c.1214del (p.Asn405Ilefs*3)
5	*USH2A*	Heterozygous	c.2276G>T (p.Cys759Phe)
5	*USH2A*	Heterozygous	c.2299del (p.Glu767Serfs*21)
6	*USH2A*	Heterozygous	c.2332G>T (p.Asp778Tyr)
6	*USH2A*	Heterozygous	c.1724G>A (p.Cys575Tyr)
7	*USH2A*	Heterozygous	c.4106C>T (p.Ser1369Leu)
7	*USH2A*	Heterozygous	c.1214del (p.Asn405Ilefs*3)
8	*USH2A*	Heterozygous	c.2276G>T (p.Cys759Phe)
8	*USH2A*	Heterozygous	c.10342G>A (p.Glu3448Lys)
9	*USH2A*	Heterozygous	c.4106C>T (p.Ser1369Leu)
9	*USH2A*	Heterozygous	c.2299del (p.Glu767Serfs*21)
10	*USH2A*	Homozygous	c.2276G>T (p.Cys759Phe)

Genotype-phenotype correlations

The median values from ophthalmic evaluation by variant are reported in Table 4. Patients with the p.Cys759Phe variant had the worst logMAR BCVA, largest VF MD, and slimmer macular thickness. When stratified by zygosity, patients with the p.Cys759Phe variant who were compound heterozygous had a median logMAR BCVA of 1.55 and 1.45 in the OD and OS, respectively. The patient who was homozygous for p.Cys759Phe had a logMAR BCVA of 2.8 and 0.4 in the OD and OS, respectively.

**Table 4:** Median values from ophthalmic evaluation by variant

Variant	VA (number, %)		VF MD (number, %)		VF PSD (number, %)		mOCT volume (number, %)		mOCT thickness (number, %)	
	OD	OS	OD	OS	OD	OS	OD	OS	OD	OS
p.Glu767Serfs*21	0.2 (4, 100)	0.2 (4, 100)	-26.1 (3, 75)	-27.2 (3, 75)	6.0 (3, 75)	6.0 (3, 75)	8.5 (4,100)	8.1 (4,100)	235 (4,100)	223 (4,100)
p.Cys575Tyr	0.2 (3, 75)	0.2 (3, 75)	-26.1 (3, 75)	-26.8 (3, 75)	7.6 (3, 75)	7.6 (3, 75)	8.4 (4,100)	8.1 (4,100)	246 (4,100)	242 (4,100)
p.Cys759Phe	2.8 (3, 100)	0.4 (3, 100)	-31.3 (2, 67)	-30.7 (2, 67)	3.8 (2, 67)	4.7 (2, 67)	5.3 (2, 67)	5.5 (2, 67)	188 (2, 67)	164 (2, 67)
p.Asn405Ilefs*3	0.2 (3, 100)	0.2 (3, 100)	-29.7 (2, 67)	-29.2 (2, 67)	4.2 (2, 67)	4.2 (2, 67)	8.3 (2, 67)	8.5 (2, 67)	231 (2, 67)	237 (2, 67)

## Discussion

The role of the p.Cys759Phe variant remains to be elucidated. It has previously been reported as one of the most commonly identified pathogenic variants for USH2, while some argue that this variant instead leads to nonsyndromic RP [8,10-13].

In our study, it was the second most common variant as it was present in three patients. Although this variant has been associated with a milder ophthalmological phenotype, in our series, patients with the p.Cys759Phe variant had the most severe ophthalmological phenotype [8,10]. They had the worst VA, worst VF MD, and slimmer macular thickness.

Previous studies have concluded that compound heterozygotes with the p.Cys759Phe variant have an earlier and more rapid progression of blindness as compared to homozygous patients [8]. In our series, the patient who had the worst logMAR BCVA results of 2.9 OD and 2.7 OS was a compound heterozygote with the p.Cys759Phe variant. Therefore, our findings agree with the results reported by Pérez-Carro et al. [8].

The p.Cys759Phe variant has been previously reported in the Spanish population, and PR was a Spanish colony for 400 years [8]. This could explain its presence in the Island’s population.

The p.Glu767Serfs*21 variant has been reported as the most commonly identified pathogenic variant for USH2 [11,14]. In our study, it was tied as the most frequent variant and was present in four patients. When compared to the p.Cys759Phe variant, the p.Glu767Serfs*21 variant has been identified as causing a more severe ophthalmologic phenotype in some studies, but not in others [10,15]. In our series, patients with the p.Cys759Phe variant had a more severe phenotype when compared to patients with the p.Glu767Serfs*21 variant.

The p.Cys575Tyr and p.Asn405Ilefs*3 variants, while less common, have previously been identified as causative for USH2 [8,16-18]. However, no attempt has been made to establish a genotype-phenotype correlation with respect to these variants. This has been difficult due to previous studies having a low sample size with regard to these variants. In our study, the p.Cys575Tyr was tied for the most common variant as it was present in four patients. Nevertheless, there was no identifiable genotype-phenotype correlation.

A limitation of our study is the small sample size since only 10 patients were found. Usher syndrome remains a rare disease. Further, some patients’ findings were not available due to the retrospective nature of the study.

## Conclusions

This study is the first report on a genotype-phenotype correlation of patients with Usher syndrome in the Puerto Rican population. Our study identified a total of seven pathogenic mutations for Usher syndrome, which are compatible with previously reported pathogenic mutations in the *USH2A* gene. In contrast to previous studies, the p.Cys759Phe variant correlated with the most severe phenotype. This variant has a high prevalence in the Spanish population, and PR was a Spanish colony for 400 years. Therefore, the presence of this variant in Puerto Rican patients could be traced back to Spain.

The genotype-phenotype correlation in these patients stresses the importance of genotyping patients with the syndrome. These patients may benefit from multispecialty management. Further studies to evaluate the common founder effect of patients with the syndrome in PR are warranted.
